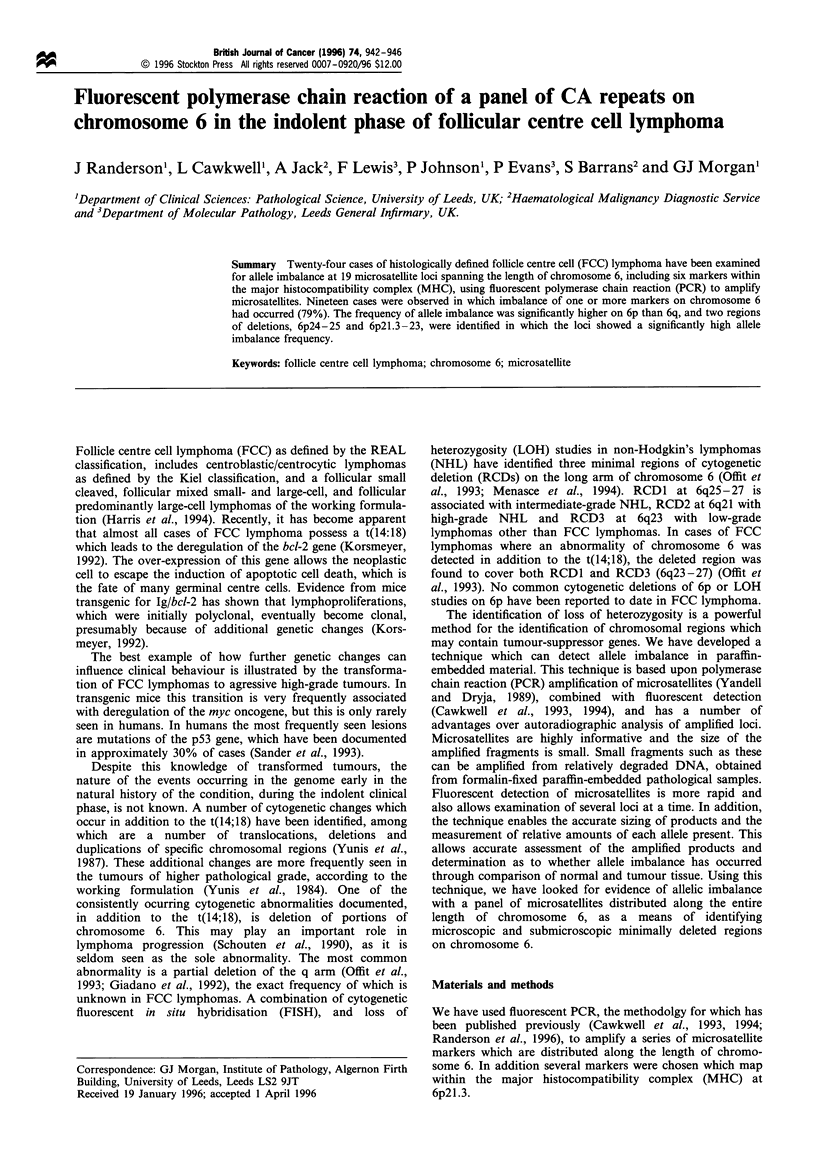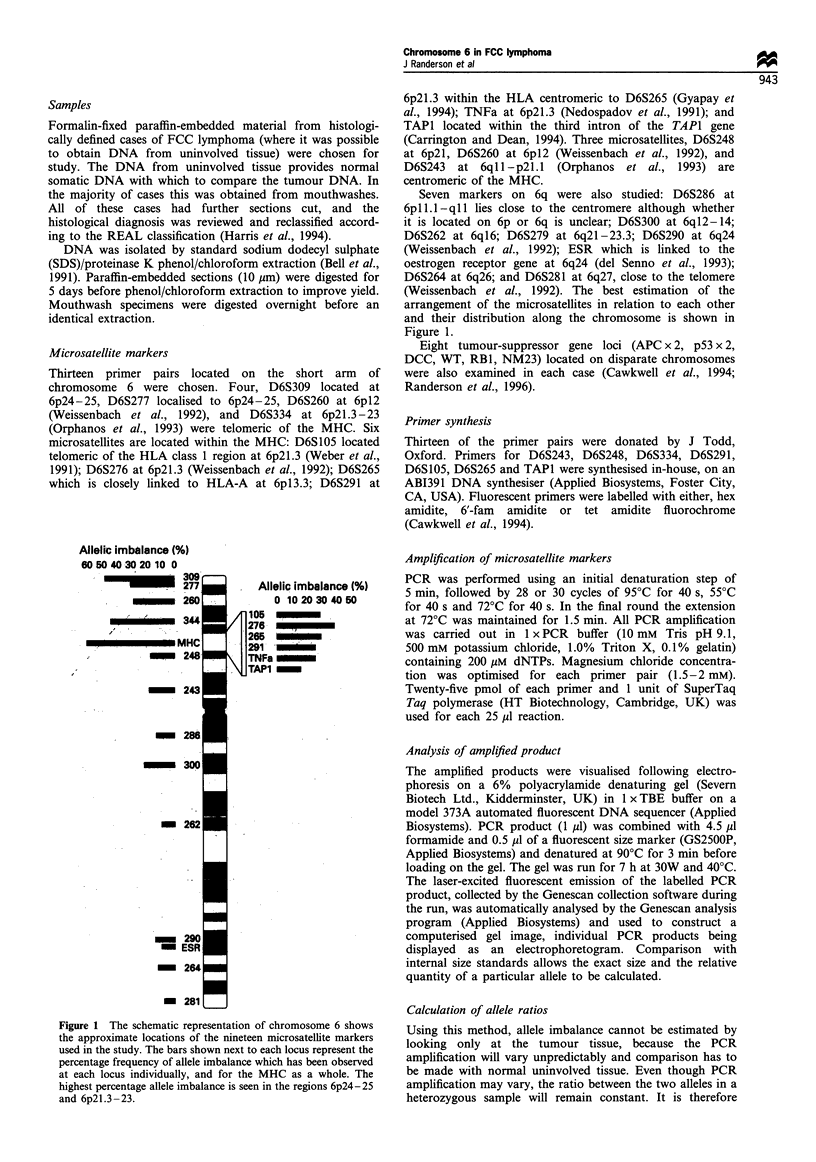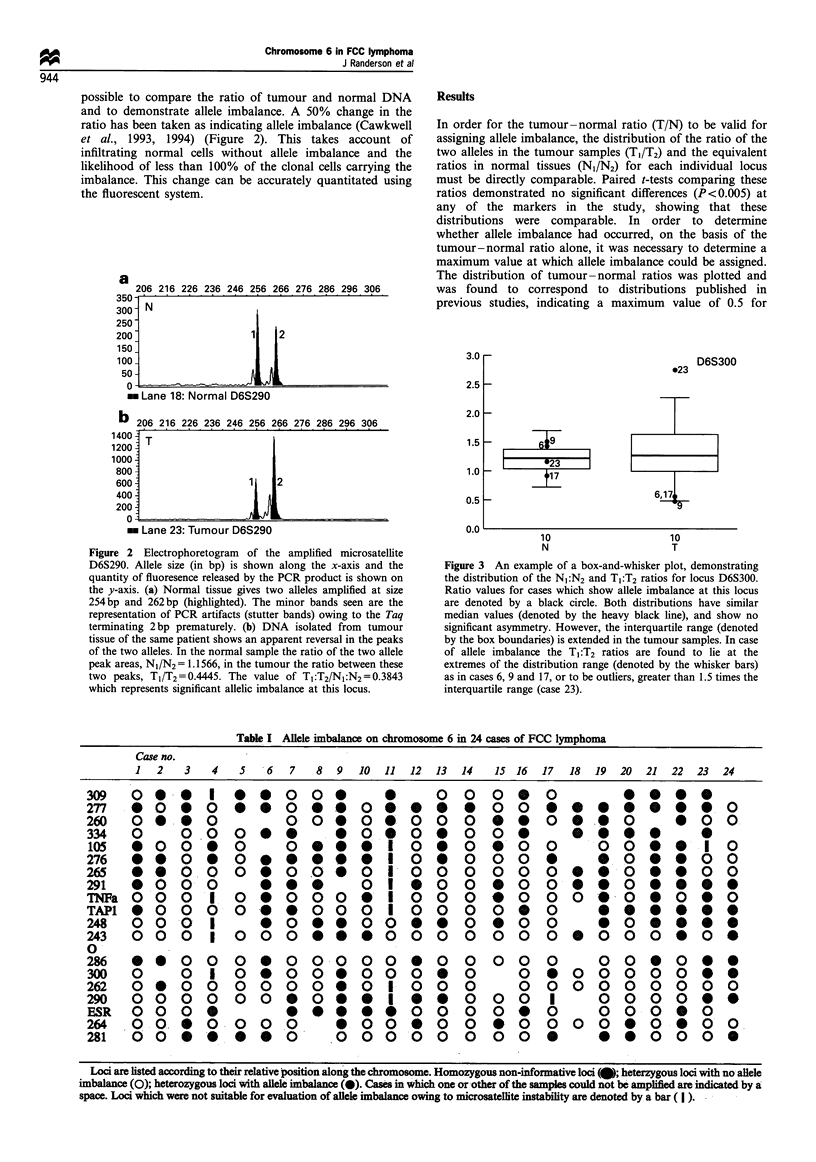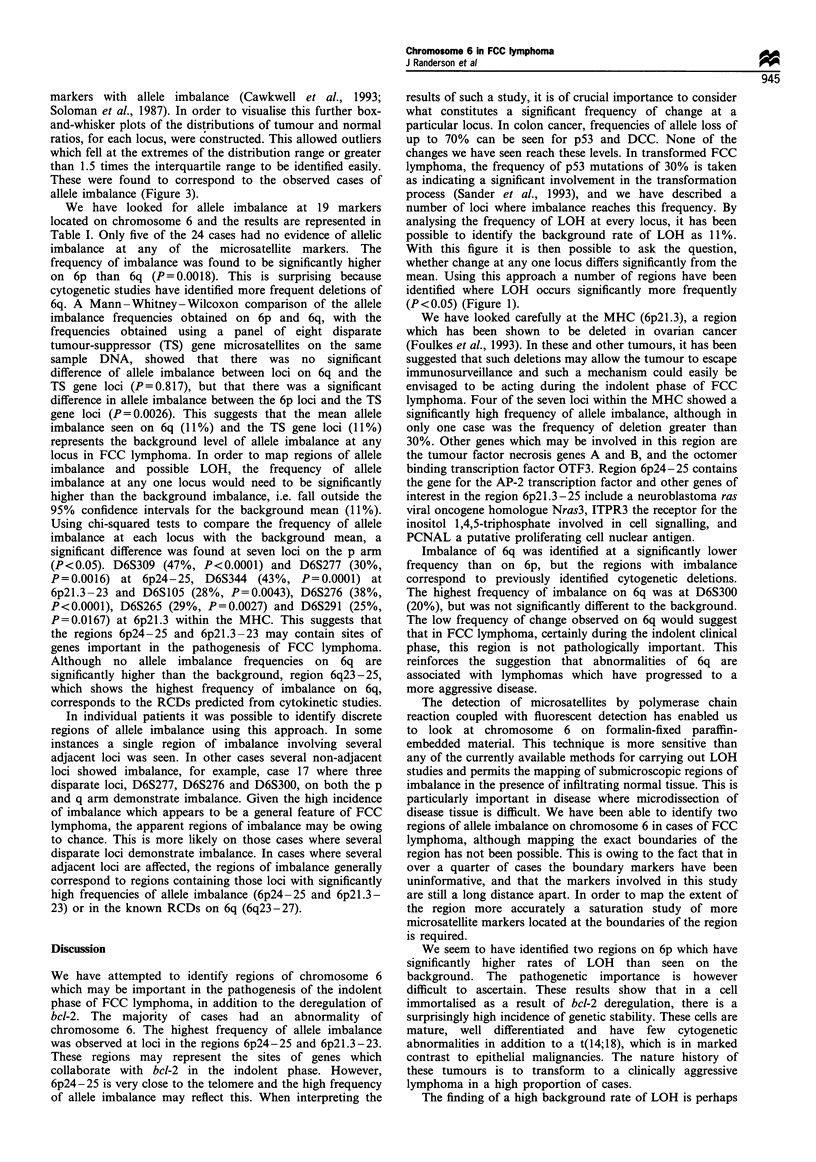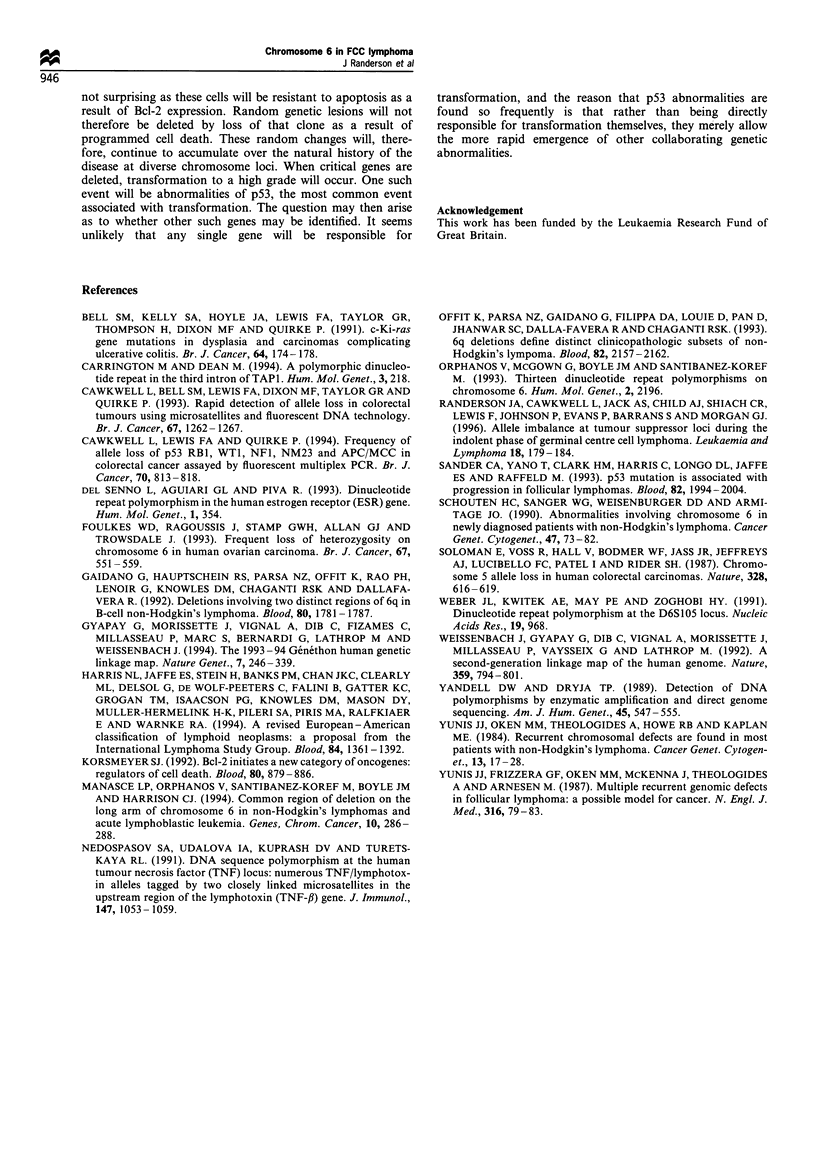# Fluorescent polymerase chain reaction of a panel of CA repeats on chromosome 6 in the indolent phase of follicular centre cell lymphoma.

**DOI:** 10.1038/bjc.1996.461

**Published:** 1996-09

**Authors:** J. Randerson, L. Cawkwell, A. Jack, F. Lewis, P. Johnson, P. Evans, S. Barrans, G. J. Morgan

**Affiliations:** Department of Clinical Sciences: Pathological Science, University of Leeds, UK.

## Abstract

Twenty-four cases of histologically defined follicle centre cell (FCC) lymphoma have been examined for allele imbalance at 19 microsatellite loci spanning the length of chromosome 6, including six markers within the major histocompatibility complex (MHC), using fluorescent polymerase chain reaction (PCR) to amplify microsatellites. Nineteen cases were observed in which imbalance of one or more markers on chromosome 6 had occurred (79%). The frequency of allele imbalance was significantly higher on 6p than 6q, and two regions of deletions, 6p24-25 and 6p21.3-23, were identified in which the loci showed a significantly high allele imbalance frequency.


British Journal of Cancer (1996) 74, 942-946
© 1996 Stockton Press All rights reserved 0007-0920/96 $12.00

### Fluorescent polymerase chain reaction of a panel of CA repeats on chromosome 6 in the indolent phase of follicular centre cell lymphoma

J Randerson[1], L Cawkwell[1], A Jack[2], F Lewis[3], P Johnson[1], P Evans[3], S Barrans[2] and GJ Morgan[1]

[1]*Department of Clinical Sciences: Pathological Science, University of Leeds, UK;* [2]*Haematological Malignancy Diagnostic Service and* [3]*Department of Molecular Pathology, Leeds General Infirmary, UK.*

**Summary** Twenty-four cases of histologically defined follicle centre cell (FCC) lymphoma have been examined for allele imbalance at 19 microsatellite loci spanning the length of chromosome 6, including six markers within the major histocompatibility complex (MHC), using fluorescent polymerase chain reaction (PCR) to amplify microsatellites. Nineteen cases were observed in which imbalance of one or more markers on chromosome 6 had occurred (79%). The frequency of allele imbalance was significantly higher on 6p than 6q, and two regions of deletions, 6p24-25 and 6p21.3-23, were identified in which the loci showed a significantly high allele imbalance frequency.

**Keywords:** follicle centre cell lymphoma; chromosome 6; microsatellite

Follicle centre cell lymphoma (FCC) as defined by the REAL classification, includes centroblastic/centrocytic lymphomas as defined by the Kiel classification, and a follicular small cleaved, follicular mixed small- and large-cell, and follicular predominantly large-cell lymphomas of the working formulation (Harris *et al.*, 1994). Recently, it has become apparent that almost all cases of FCC lymphoma possess a t(14:18) which leads to the deregulation of the *bcl*-2 gene (Korsmeyer, 1992). The over-expression of this gene allows the neoplastic cell to escape the induction of apoptotic cell death, which is the fate of many germinal centre cells. Evidence from mice transgenic for Ig/*bcl*-2 has shown that lymphoproliferations, which were initially polyclonal, eventually become clonal, presumably because of additional genetic changes (Korsmeyer, 1992).

The best example of how further genetic changes can influence clinical behaviour is illustrated by the transformation of FCC lymphomas to agressive high-grade tumours. In transgenic mice this transition is very frequently associated with deregulation of the *myc* oncogene, but this is only rarely seen in humans. In humans the most frequently seen lesions are mutations of the p53 gene, which have been documented in approximately 30% of cases (Sander *et al.*, 1993).

Despite this knowledge of transformed tumours, the nature of the events occurring in the genome early in the natural history of the condition, during the indolent clinical phase, is not known. A number of cytogenetic changes which occur in addition to the t(14;18) have been identified, among which are a number of translocations, deletions and duplications of specific chromosomal regions (Yunis *et al.*, 1987). These additional changes are more frequently seen in the tumours of higher pathological grade, according to the working formulation (Yunis *et al.*, 1984). One of the consistently ocurring cytogenetic abnormalities documented, in addition to the t(14;18), is deletion of portions of chromosome 6. This may play an important role in lymphoma progression (Schouten *et al.*, 1990), as it is seldom seen as the sole abnormality. The most common abnormality is a partial deletion of the q arm (Offit *et al.*, 1993; Giadano *et al.*, 1992), the exact frequency of which is unknown in FCC lymphomas. A combination of cytogenetic fluorescent *in situ* hybridisation (FISH), and loss of heterozygosity (LOH) studies in non-Hodgkin's lymphomas (NHL) have identified three minimal regions of cytogenetic deletion (RCDs) on the long arm of chromosome 6 (Offit *et al.*, 1993; Menasce *et al.*, 1994). RCD1 at 6q25-27 is associated with intermediate-grade NHL, RCD2 at 6q21 with high-grade NHL and RCD3 at 6q23 with low-grade lymphomas other than FCC lymphomas. In cases of FCC lymphomas where an abnormality of chromosome 6 was detected in addition to the t(14;18), the deleted region was found to cover both RCD1 and RCD3 (6q23-27) (Offit *et al.*, 1993). No common cytogenetic deletions of 6p or LOH studies on 6p have been reported to date in FCC lymphoma.

The identification of loss of heterozygosity is a powerful method for the identification of chromosomal regions which may contain tumour-suppressor genes. We have developed a technique which can detect allele imbalance in paraffin-embedded material. This technique is based upon polymerase chain reaction (PCR) amplification of microsatellites (Yandell and Dryja, 1989), combined with fluorescent detection (Cawkwell *et al.*, 1993, 1994), and has a number of advantages over autoradiographic analysis of amplified loci. Microsatellites are highly informative and the size of the amplified fragments is small. Small fragments such as these can be amplified from relatively degraded DNA, obtained from formalin-fixed paraffin-embedded pathological samples. Fluorescent detection of microsatellites is more rapid and also allows examination of several loci at a time. In addition, the technique enables the accurate sizing of products and the measurement of relative amounts of each allele present. This allows accurate assessment of the amplified products and determination as to whether allele imbalance has occurred through comparison of normal and tumour tissue. Using this technique, we have looked for evidence of allelic imbalance with a panel of microsatellites distributed along the entire length of chromosome 6, as a means of identifying microscopic and submicroscopic minimally deleted regions on chromosome 6.

### Materials and methods

We have used fluorescent PCR, the methodolgy for which has been published previously (Cawkwell *et al.*, 1993, 1994; Randerson *et al.*, 1996), to amplify a series of microsatellite markers which are distributed along the length of chromosome 6. In addition several markers were chosen which map within the major histocompatibility complex (MHC) at 6p21.3.

Correspondence: GJ Morgan, Institute of Pathology, Algernon Firth Building, University of Leeds, Leeds LS2 9JT
Received 19 January 1996; accepted 1 April 1996

*Samples*

Formalin-fixed paraffin-embedded material from histologically defined cases of FCC lymphoma (where it was possible to obtain DNA from uninvolved tissue) were chosen for study. The DNA from uninvolved tissue provides normal somatic DNA with which to compare the tumour DNA. In the majority of cases this was obtained from mouthwashes. All of these cases had further sections cut, and the histological diagnosis was reviewed and reclassified according to the REAL classification (Harris *et al.*, 1994).

DNA was isolated by standard sodium dodecyl sulphate (SDS)/proteinase K phenol/chloroform extraction (Bell *et al.*, 1991). Paraffin-embedded sections (10 $\mu$m) were digested for 5 days before phenol/chloroform extraction to improve yield. Mouthwash specimens were digested overnight before an identical extraction.

*Microsatellite markers*

Thirteen primer pairs located on the short arm of chromosome 6 were chosen. Four, D6S309 located at 6p24–25, D6S277 localised to 6p24–25, D6S260 at 6p12 (Weissenbach *et al.*, 1992), and D6S334 at 6p21.3–23 (Orphanos *et al.*, 1993) were telomeric of the MHC. Six microsatellites are located within the MHC: D6S105 located telomeric of the HLA class 1 region at 6p21.3 (Weber *et al.*, 1991); D6S276 at 6p21.3 (Weissenbach *et al.*, 1992); D6S265 which is closely linked to HLA-A at 6p13.3; D6S291 at 6p21.3 within the HLA centromeric to D6S265 (Gyapay *et al.*, 1994); TNFa at 6p21.3 (Nedospadov *et al.*, 1991); and TAP1 located within the third intron of the *TAP*1 gene (Carrington and Dean, 1994). Three microsatellites, D6S248 at 6p21, D6S260 at 6p12 (Weissenbach *et al.*, 1992), and D6S243 at 6q11–p21.1 (Orphanos *et al.*, 1993) are centromeric of the MHC.

Seven markers on 6q were also studied: D6S286 at 6p11.1–q11 lies close to the centromere although whether it is located on 6p or 6q is unclear; D6S300 at 6q12–14; D6S262 at 6q16; D6S279 at 6q21–23.3; D6S290 at 6q24 (Weissenbach *et al.*, 1992); ESR which is linked to the oestrogen receptor gene at 6q24 (del Senno *et al.*, 1993); D6S264 at 6q26; and D6S281 at 6q27, close to the telomere (Weissenbach *et al.*, 1992). The best estimation of the arrangement of the microsatellites in relation to each other and their distribution along the chromosome is shown in Figure 1.

Eight tumour-suppressor gene loci (APC × 2, p53 × 2, DCC, WT, RB1, NM23) located on disparate chromosomes were also examined in each case (Cawkwell *et al.*, 1994; Randerson *et al.*, 1996).

**Figure 1** The schematic representation of chromosome 6 shows the approximate locations of the nineteen microsatellite markers used in the study. The bars shown next to each locus represent the percentage frequency of allele imbalance which has been observed at each locus individually, and for the MHC as a whole. The highest percentage allele imbalance is seen in the regions 6p24–25 and 6p21.3–23.

*Primer synthesis*

Thirteen of the primer pairs were donated by J Todd, Oxford. Primers for D6S243, D6S248, D6S334, D6S291, D6S105, D6S265 and TAP1 were synthesised in-house, on an ABI391 DNA synthesiser (Applied Biosystems, Foster City, CA, USA). Fluorescent primers were labelled with either, hex amidite, 6′-fam amidite or tet amidite fluorochrome (Cawkwell *et al.*, 1994).

*Amplification of microsatellite markers*

PCR was performed using an initial denaturation step of 5 min, followed by 28 or 30 cycles of 95°C for 40 s, 55°C for 40 s and 72°C for 40 s. In the final round the extension at 72°C was maintained for 1.5 min. All PCR amplification was carried out in 1 × PCR buffer (10 mM Tris pH 9.1, 500 mM potassium chloride, 1.0% Triton X, 0.1% gelatin) containing 200 $\mu$M dNTPs. Magnesium chloride concentration was optimised for each primer pair (1.5–2 mM). Twenty-five pmol of each primer and 1 unit of SuperTaq *Taq* polymerase (HT Biotechnology, Cambridge, UK) was used for each 25 $\mu$l reaction.

*Analysis of amplified product*

The amplified products were visualised following electrophoresis on a 6% polyacrylamide denaturing gel (Severn Biotech Ltd., Kidderminster, UK) in 1 × TBE buffer on a model 373A automated fluorescent DNA sequencer (Applied Biosystems). PCR product (1 $\mu$l) was combined with 4.5 $\mu$l formamide and 0.5 $\mu$l of a fluorescent size marker (GS2500P, Applied Biosystems) and denatured at 90°C for 3 min before loading on the gel. The gel was run for 7 h at 30W and 40°C. The laser-excited fluorescent emission of the labelled PCR product, collected by the Genescan collection software during the run, was automatically analysed by the Genescan analysis program (Applied Biosystems) and used to construct a computerised gel image, individual PCR products being displayed as an electrophoretogram. Comparison with internal size standards allows the exact size and the relative quantity of a particular allele to be calculated.

*Calculation of allele ratios*

Using this method, allele imbalance cannot be estimated by looking only at the tumour tissue, because the PCR amplification will vary unpredictably and comparison has to be made with normal uninvolved tissue. Even though PCR amplification may vary, the ratio between the two alleles in a heterozygous sample will remain constant. It is therefore possible to compare the ratio of tumour and normal DNA and to demonstrate allele imbalance. A 50% change in the ratio has been taken as indicating allele imbalance (Cawkwell *et al.*, 1993, 1994) (Figure 2). This takes account of infiltrating normal cells without allele imbalance and the likelihood of less than 100% of the clonal cells carrying the imbalance. This change can be accurately quantitated using the fluorescent system.

**Figure 2** Electrophoretogram of the amplified microsatellite D6S290. Allele size (in bp) is shown along the *x*-axis and the quantity of fluoresence released by the PCR product is shown on the *y*-axis. (**a**) Normal tissue gives two alleles amplified at size 254 bp and 262 bp (highlighted). The minor bands seen are the representation of PCR artifacts (stutter bands) owing to the *Taq* terminating 2 bp prematurely. (**b**) DNA isolated from tumour tissue of the same patient shows an apparent reversal in the peaks of the two alleles. In the normal sample the ratio of the two allele peak areas, $N_1/N_2 = 1.1566$, in the tumour the ratio between these two peaks, $T_1/T_2 = 0.4445$. The value of $T_1{:}T_2/N_1{:}N_2 = 0.3843$ which represents significant allelic imbalance at this locus.

### Results

In order for the tumour–normal ratio (T/N) to be valid for assigning allele imbalance, the distribution of the ratio of the two alleles in the tumour samples ($T_1/T_2$) and the equivalent ratios in normal tissues ($N_1/N_2$) for each individual locus must be directly comparable. Paired *t*-tests comparing these ratios demonstrated no significant differences ($P < 0.005$) at any of the markers in the study, showing that these distributions were comparable. In order to determine whether allele imbalance had occurred, on the basis of the tumour–normal ratio alone, it was necessary to determine a maximum value at which allele imbalance could be assigned. The distribution of tumour–normal ratios was plotted and was found to correspond to distributions published in previous studies, indicating a maximum value of 0.5 for

**Figure 3** An example of a box-and-whisker plot, demonstrating the distribution of the $N_1{:}N_2$ and $T_1{:}T_2$ ratios for locus D6S300. Ratio values for cases which show allele imbalance at this locus are denoted by a black circle. Both distributions have similar median values (denoted by the heavy black line), and show no significant asymmetry. However, the interquartile range (denoted by the box boundaries) is extended in the tumour samples. In case of allele imbalance the $T_1{:}T_2$ ratios are found to lie at the extremes of the distribution range (denoted by the whisker bars) as in cases 6, 9 and 17, or to be outliers, greater than 1.5 times the interquartile range (case 23).

**Table I** Allele imbalance on chromosome 6 in 24 cases of FCC lymphoma

| *Case no.* | *1* | *2* | *3* | *4* | *5* | *6* | *7* | *8* | *9* | *10* | *11* | *12* | *13* | *14* | *15* | *16* | *17* | *18* | *19* | *20* | *21* | *22* | *23* | *24* |
|---|---|---|---|---|---|---|---|---|---|---|---|---|---|---|---|---|---|---|---|---|---|---|---|---|
| 309 | ○ | ● | ● | \| | ◍ | ● | ○ | ○ | ● |  | ● |  | ○ | ○ | ○ | ◍ | ○ |  |  | ● | ◍ | ● | ◍ |  |
| 277 | ◍ | ○ | ◍ | ○ | ◍ | ◍ | ○ | ◍ | ◍ | ○ | ◍ | ◍ | ◍ | ◍ | ○ | ○ | ● | ◍ | ◍ | ◍ | ◍ | ● | ● | ○ |
| 260 | ○ | ● | ◍ | ○ |  |  | ○ | ○ | ◍ | ○ | ● | ○ | ○ | ○ | ◍ | ● | ○ | ◍ | ◍ | ○ |  | ● | ○ | ○ |
| 334 | ○ |  | ○ | ○ | ○ | ● | ◍ |  | ● | ○ | ◍ | ○ | ● | ○ | ○ | ● |  | ◍ | ● | ◍ | ● |  | ◍ |  |
| 105 | ● | ○ | ○ | ● | ○ |  | ○ | ◍ | ● | ◍ | \| | ○ | ● | ○ | ◍ | ○ | ○ |  | ○ | ○ | ● | ◍ | \| | ○ |
| 276 | ● | ● | ○ | ● | ○ | ◍ | ◍ | ◍ | ◍ | ◍ | \| | ○ | ● | ○ | ○ | ○ | ● |  | ◍ | ○ | ◍ | ● | ○ | ○ |
| 265 | ● | ◍ | ○ | ○ | ○ | ● | ○ | ○ | ● | ○ | \| | ○ | ○ | ○ | ○ | ○ | ○ | ◍ | ◍ | ○ | ● | ● | ○ | ○ |
| 291 | ◍ | ○ | ○ | ○ |  | ● | ◍ | ◍ |  | ○ | \| | ◍ | ○ | ○ | ◍ | ○ | ○ | ◍ | ◍ | ○ | ● | ◍ | ◍ | ● |
| TNFa | ○ | ○ | ○ | \| | ○ | ● | ○ | ○ | ○ | ● | \| | ○ | ○ | ○ | ● | ○ | ○ | ○ | ◍ | ○ | ● | ○ | ● | ○ |
| TAP1 | ◍ | ○ | ○ | ○ | ○ | ◍ | ◍ | ○ | ○ | ○ | \| | ○ | ○ | ○ | ○ | ◍ | ○ |  | ◍ | ◍ | ● | ● | ◍ | ◍ |
| 248 | ○ | ○ | ○ | \| |  | ● | ○ | ◍ | ◍ | ○ | ○ | ◍ | ● | ○ | ◍ | ○ | ○ |  | ◍ | ○ | ◍ | ◍ | ◍ | ◍ |
| 243<br>O | ○ | ○ | ○ | \| | ○ | ○ | ○ | ● | ◍ | ● | ○ | ○ | ○ | ○ | ○ | ○ | ○ | ◍ | ○ | ○ | ○ | ● | ○ | ● |
| 286 | ◍ | ◍ | ○ | ○ | ○ | ◍ | ○ | ○ | ○ | ○ | ○ | ◍ | ○ | ○ | ○ | ○ | ○ |  | ○ | ○ | ◍ | ○ | ● | ● |
| 300 | ○ |  | ○ | \| | ○ | ● | ○ | ○ | ● | ○ | ○ | ○ | ◍ | ○ |  | ○ | ● | ○ | ○ | ○ | ○ | ○ | ● | ◍ |
| 262 | ○ | ● | ○ | ○ | ○ | ○ | ○ | ○ | ● | ○ | \| | ○ | ○ | ○ |  | ○ | ○ | ○ | ○ | ○ | ○ | ○ | ○ | ○ |
| 290 | ○ | ○ | ○ | ○ | ○ | ○ | ◍ | ○ | ● | ◍ | \| | ◍ | ◍ | ○ | ○ | ○ | \| |  | ○ | ○ | ○ | ○ | ◍ | ● |
| ESR | ○ | ○ | ○ | ◍ |  |  | ◍ | ◍ | ◍ | ◍ | ◍ | ○ | ○ | ○ | ○ | ● | ○ |  | ○ | ○ | ○ | ◍ | ○ |  |
| 264 | ○ | ○ | ◍ | ○ | ○ | ○ | ○ |  | ● | ○ | ○ | ◍ | ○ | ○ | ● | ○ | ○ | ○ | ○ | ◍ | ○ | ◍ | ○ | ○ |
| 281 | ○ | ○ | ◍ | ◍ | ◍ | ◍ | ○ |  | ○ | ○ | ○ | ○ | ○ | ○ | ○ | ○ | ◍ |  | ◍ | ● | ○ | ○ | ○ | ◍ |

Loci are listed according to their relative position along the chromosome. Homozygous non-informative loci (◍); heterzygous loci with no allele imbalance (○); heterozygous loci with allele imbalance (●). Cases in which one or other of the samples could not be amplified are indicated by a space. Loci which were not suitable for evaluation of allele imbalance owing to microsatellite instability are denoted by a bar ( | ).

markers with allele imbalance (Cawkwell *et al*., 1993; Soloman *et al*., 1987). In order to visualise this further box-and-whisker plots of the distributions of tumour and normal ratios, for each locus, were constructed. This allowed outliers which fell at the extremes of the distribution range or greater than 1.5 times the interquartile range to be identified easily. These were found to correspond to the observed cases of allele imbalance (Figure 3).

We have looked for allele imbalance at 19 markers located on chromosome 6 and the results are represented in Table I. Only five of the 24 cases had no evidence of allelic imbalance at any of the microsatellite markers. The frequency of imbalance was found to be significantly higher on 6p than 6q ($P=0.0018$). This is surprising because cytogenetic studies have identified more frequent deletions of 6q. A Mann–Whitney–Wilcoxon comparison of the allele imbalance frequencies obtained on 6p and 6q, with the frequencies obtained using a panel of eight disparate tumour-suppressor (TS) gene microsatellites on the same sample DNA, showed that there was no significant difference of allele imbalance between loci on 6q and the TS gene loci ($P=0.817$), but that there was a significant difference in allele imbalance between the 6p loci and the TS gene loci ($P=0.0026$). This suggests that the mean allele imbalance seen on 6q (11%) and the TS gene loci (11%) represents the background level of allele imbalance at any locus in FCC lymphoma. In order to map regions of allele imbalance and possible LOH, the frequency of allele imbalance at any one locus would need to be significantly higher than the background imbalance, i.e. fall outside the 95% confidence intervals for the background mean (11%). Using chi-squared tests to compare the frequency of allele imbalance at each locus with the background mean, a significant difference was found at seven loci on the p arm ($P<0.05$). D6S309 (47%, $P<0.0001$) and D6S277 (30%, $P=0.0016$) at 6p24–25, D6S344 (43%, $P=0.0001$) at 6p21.3–23 and D6S105 (28%, $P=0.0043$), D6S276 (38%, $P<0.0001$), D6S265 (29%, $P=0.0027$) and D6S291 (25%, $P=0.0167$) at 6p21.3 within the MHC. This suggests that the regions 6p24–25 and 6p21.3–23 may contain sites of genes important in the pathogenesis of FCC lymphoma. Although no allele imbalance frequencies on 6q are significantly higher than the background, region 6q23–25, which shows the highest frequency of imbalance on 6q, corresponds to the RCDs predicted from cytokinetic studies.

In individual patients it was possible to identify discrete regions of allele imbalance using this approach. In some instances a single region of imbalance involving several adjacent loci was seen. In other cases several non-adjacent loci showed imbalance, for example, case 17 where three disparate loci, D6S277, D6S276 and D6S300, on both the p and q arm demonstrate imbalance. Given the high incidence of imbalance which appears to be a general feature of FCC lymphoma, the apparent regions of imbalance may be owing to chance. This is more likely on those cases where several disparate loci demonstrate imbalance. In cases where several adjacent loci are affected, the regions of imbalance generally correspond to regions containing those loci with significantly high frequencies of allele imbalance (6p24–25 and 6p21.3–23) or in the known RCDs on 6q (6q23–27).

### Discussion

We have attempted to identify regions of chromosome 6 which may be important in the pathogenesis of the indolent phase of FCC lymphoma, in addition to the deregulation of *bcl*-2. The majority of cases had an abnormality of chromosome 6. The highest frequency of allele imbalance was observed at loci in the regions 6p24–25 and 6p21.3–23. These regions may represent the sites of genes which collaborate with *bcl*-2 in the indolent phase. However, 6p24–25 is very close to the telomere and the high frequency of allele imbalance may reflect this. When interpreting the results of such a study, it is of crucial importance to consider what constitutes a significant frequency of change at a particular locus. In colon cancer, frequencies of allele loss of up to 70% can be seen for p53 and DCC. None of the changes we have seen reach these levels. In transformed FCC lymphoma, the frequency of p53 mutations of 30% is taken as indicating a significant involvement in the transformation process (Sander *et al*., 1993), and we have described a number of loci where imbalance reaches this frequency. By analysing the frequency of LOH at every locus, it has been possible to identify the background rate of LOH as 11%. With this figure it is then possible to ask the question, whether change at any one locus differs significantly from the mean. Using this approach a number of regions have been identified where LOH occurs significantly more frequently ($P<0.05$) (Figure 1).

We have looked carefully at the MHC (6p21.3), a region which has been shown to be deleted in ovarian cancer (Foulkes *et al*., 1993). In these and other tumours, it has been suggested that such deletions may allow the tumour to escape immunosurveillance and such a mechanism could easily be envisaged to be acting during the indolent phase of FCC lymphoma. Four of the seven loci within the MHC showed a significantly high frequency of allele imbalance, although in only one case was the frequency of deletion greater than 30%. Other genes which may be involved in this region are the tumour factor necrosis genes A and B, and the octomer binding transcription factor OTF3. Region 6p24–25 contains the gene for the AP-2 transcription factor and other genes of interest in the region 6p21.3–25 include a neuroblastoma *ras* viral oncogene homologue N*ras*3, ITPR3 the receptor for the inositol 1,4,5-triphosphate involved in cell signalling, and PCNAL a putative proliferating cell nuclear antigen.

Imbalance of 6q was identified at a significantly lower frequency than on 6p, but the regions with imbalance correspond to previously identified cytogenetic deletions. The highest frequency of imbalance on 6q was at D6S300 (20%), but was not significantly different to the background. The low frequency of change observed on 6q would suggest that in FCC lymphoma, certainly during the indolent clinical phase, this region is not pathologically important. This reinforces the suggestion that abnormalities of 6q are associated with lymphomas which have progressed to a more aggressive disease.

The detection of microsatellites by polymerase chain reaction coupled with fluorescent detection has enabled us to look at chromosome 6 on formalin-fixed paraffin-embedded material. This technique is more sensitive than any of the currently available methods for carrying out LOH studies and permits the mapping of submicroscopic regions of imbalance in the presence of infiltrating normal tissue. This is particularly important in disease where microdissection of disease tissue is difficult. We have been able to identify two regions of allele imbalance on chromosome 6 in cases of FCC lymphoma, although mapping the exact boundaries of the region has not been possible. This is owing to the fact that in over a quarter of cases the boundary markers have been uninformative, and that the markers involved in this study are still a long distance apart. In order to map the extent of the region more accurately a saturation study of more microsatellite markers located at the boundaries of the region is required.

We seem to have identified two regions on 6p which have significantly higher rates of LOH than seen on the background. The pathogenetic importance is however difficult to ascertain. These results show that in a cell immortalised as a result of *bcl*-2 deregulation, there is a surprisingly high incidence of genetic stability. These cells are mature, well differentiated and have few cytogenetic abnormalities in addition to a t(14;18), which is in marked contrast to epithelial malignancies. The nature history of these tumours is to transform to a clinically aggressive lymphoma in a high proportion of cases.

The finding of a high background rate of LOH is perhaps not surprising as these cells will be resistant to apoptosis as a result of Bcl-2 expression. Random genetic lesions will not therefore be deleted by loss of that clone as a result of programmed cell death. These random changes will, therefore, continue to accumulate over the natural history of the disease at diverse chromosome loci. When critical genes are deleted, transformation to a high grade will occur. One such event will be abnormalities of p53, the most common event associated with transformation. The question may then arise as to whether other such genes may be identified. It seems unlikely that any single gene will be responsible for transformation, and the reason that p53 abnormalities are found so frequently is that rather than being directly responsible for transformation themselves, they merely allow the more rapid emergence of other collaborating genetic abnormalities.

**Acknowledgement**
This work has been funded by the Leukaemia Research Fund of Great Britain.

### References

BELL SM, KELLY SA, HOYLE JA, LEWIS FA, TAYLOR GR, THOMPSON H, DIXON MF AND QUIRKE P. (1991). c-Ki-*ras* gene mutations in dysplasia and carcinomas complicating ulcerative colitis. *Br. J. Cancer*, **64**, 174–178.

CARRINGTON M AND DEAN M. (1994). A polymorphic dinucleotide repeat in the third intron of TAP1. *Hum. Mol. Genet.*, **3**, 218.

CAWKWELL L, BELL SM, LEWIS FA, DIXON MF, TAYLOR GR AND QUIRKE P. (1993). Rapid detection of allele loss in colorectal tumours using microsatellites and fluorescent DNA technology. *Br. J. Cancer*, **67**, 1262–1267.

CAWKWELL L, LEWIS FA AND QUIRKE P. (1994). Frequency of allele loss of p53 RB1, WT1, NF1, NM23 and APC/MCC in colorectal cancer assayed by fluorescent multiplex PCR. *Br. J. Cancer*, **70**, 813–818.

DEL SENNO L, AGUIARI GL AND PIVA R. (1993). Dinucleotide repeat polymorphism in the human estrogen receptor (ESR) gene. *Hum. Mol. Genet.*, **1**, 354.

FOULKES WD, RAGOUSSIS J, STAMP GWH, ALLAN GJ AND TROWSDALE J. (1993). Frequent loss of heterozygosity on chromosome 6 in human ovarian carcinoma. *Br. J. Cancer*, **67**, 551–559.

GAIDANO G, HAUPTSCHEIN RS, PARSA NZ, OFFIT K, RAO PH, LENOIR G, KNOWLES DM, CHAGANTI RSK AND DALLAFAVERA R. (1992). Deletions involving two distinct regions of 6q in B-cell non-Hodgkin's lymphoma. *Blood*, **80**, 1781–1787.

GYAPAY G, MORISSETTE J, VIGNAL A, DIB C, FIZAMES C, MILLASSEAU P, MARC S, BERNARDI G, LATHROP M AND WEISSENBACH J. (1994). The 1993–94 Généthon human genetic linkage map. *Nature Genet.*, **7**, 246–339.

HARRIS NL, JAFFE ES, STEIN H, BANKS PM, CHAN JKC, CLEARLY ML, DELSOL G, DE WOLF-PEETERS C, FALINI B, GATTER KC, GROGAN TM, ISAACSON PG, KNOWLES DM, MASON DY, MULLER-HERMELINK H-K, PILERI SA, PIRIS MA, RALFKIAER E AND WARNKE RA. (1994). A revised European–American classification of lymphoid neoplasms: a proposal from the International Lymphoma Study Group. *Blood*, **84**, 1361–1392.

KORSMEYER SJ. (1992). Bcl-2 initiates a new category of oncogenes: regulators of cell death. *Blood*, **80**, 879–886.

MANASCE LP, ORPHANOS V, SANTIBANEZ-KOREF M, BOYLE JM AND HARRISON CJ. (1994). Common region of deletion on the long arm of chromosome 6 in non-Hodgkin's lymphomas and acute lymphoblastic leukemia. *Genes, Chrom. Cancer*, **10**, 286–288.

NEDOSPASOV SA, UDALOVA IA, KUPRASH DV AND TURETSKAYA RL. (1991). DNA sequence polymorphism at the human tumour necrosis factor (TNF) locus: numerous TNF/lymphotoxin alleles tagged by two closely linked microsatellites in the upstream region of the lymphotoxin (TNF-$\beta$) gene. *J. Immunol.*, **147**, 1053–1059.

OFFIT K, PARSA NZ, GAIDANO G, FILIPPA DA, LOUIE D, PAN D, JHANWAR SC, DALLA-FAVERA R AND CHAGANTI RSK. (1993). 6q deletions define distinct clinicopathologic subsets of non-Hodgkin's lympoma. *Blood*, **82**, 2157–2162.

ORPHANOS V, MCGOWN G, BOYLE JM AND SANTIBANEZ-KOREF M. (1993). Thirteen dinucleotide repeat polymorphisms on chromosome 6. *Hum. Mol. Genet.*, **2**, 2196.

RANDERSON JA, CAWKWELL L, JACK AS, CHILD AJ, SHIACH CR, LEWIS F, JOHNSON P, EVANS P, BARRANS S AND MORGAN GJ. (1996). Allele imbalance at tumour suppressor loci during the indolent phase of germinal centre cell lymphoma. *Leukaemia and Lymphoma* **18**, 179–184.

SANDER CA, YANO T, CLARK HM, HARRIS C, LONGO DL, JAFFE ES AND RAFFELD M. (1993). p53 mutation is associated with progression in follicular lymphomas. *Blood*, **82**, 1994–2004.

SCHOUTEN HC, SANGER WG, WEISENBURGER DD AND ARMITAGE JO. (1990). Abnormalities involving chromosome 6 in newly diagnosed patients with non-Hodgkin's lymphoma. *Cancer Genet. Cytogenet.*, **47**, 73–82.

SOLOMAN E, VOSS R, HALL V, BODMER WF, JASS JR, JEFFREYS AJ, LUCIBELLO FC, PATEL I AND RIDER SH. (1987). Chromosome 5 allele loss in human colorectal carcinomas. *Nature*, **328**, 616–619.

WEBER JL, KWITEK AE, MAY PE AND ZOGHOBI HY. (1991). Dinucleotide repeat polymorphism at the D6S105 locus. *Nucleic Acids Res.*, **19**, 968.

WEISSENBACH J, GYAPAY G, DIB C, VIGNAL A, MORISSETTE J, MILLASSEAU P, VAYSSEIX G AND LATHROP M. (1992). A second-generation linkage map of the human genome. *Nature*, **359**, 794–801.

YANDELL DW AND DRYJA TP. (1989). Detection of DNA polymorphisms by enzymatic amplification and direct genome sequencing. *Am. J. Hum. Genet.*, **45**, 547–555.

YUNIS JJ, OKEN MM, THEOLOGIDES A, HOWE RB AND KAPLAN ME. (1984). Recurrent chromosomal defects are found in most patients with non-Hodgkin's lymphoma. *Cancer Genet. Cytogenet.*, **13**, 17–28.

YUNIS JJ, FRIZZERA GF, OKEN MM, MCKENNA J, THEOLOGIDES A AND ARNESEN M. (1987). Multiple recurrent genomic defects in follicular lymphoma: a possible model for cancer. *N. Engl. J. Med.*, **316**, 79–83.